# Stakeholder-Guided Formation of a Statewide Community Pharmacy Practice-Based Research Network

**DOI:** 10.3390/pharmacy7030118

**Published:** 2019-08-17

**Authors:** Joni C. Carroll, Melissa Somma McGivney, Kim C. Coley

**Affiliations:** School of Pharmacy, University of Pittsburgh, Pittsburgh, PA 15260, USA

**Keywords:** practice-based research networks, community pharmacy research networks, patient-centered outcomes research, community pharmacy, pharmacists, research, clinically-integrated network

## Abstract

Community pharmacies across the United States are forming clinically integrated networks (CINs) to facilitate the provision of patient-centered care. These networks need to continually innovate and demonstrate how their patient care services positively impact health outcomes. One way to do this is to develop a practice-based research network (PBRN) in partnership with existing CINs to perform robust outcome evaluations. The objective of this study was to learn pharmacists’ opinions on participating in research to facilitate the formation of a community pharmacy PBRN in Pennsylvania. A 20-item survey gathered information on pharmacists’ research interests, perceived benefits of research participation, and preferences on communication and patient engagement. Descriptive statistics and Chi-square tests were used to analyze quantitative data. Seventy-three participants completed the survey, with 47% representing independent pharmacies. The majority (96%) were interested in research opportunities and 86% believed improving workflow and patient care was the most valuable benefit. Eighty percent of pharmacists reported it is very important to demonstrate that pharmacists care about making patients’ health better. Connecting pharmacists with other health care providers was reported as very important by 75% of respondents. Pharmacists reported face-to-face communication (76%) as their preferred way to approach patients about research and 72% supported using student pharmacists to assist with patient engagement. The results from this study can inform others who are structuring processes and developing communication strategies for community pharmacy PBRNs, particularly in partnership with CINs.

## 1. Introduction

The Centers for Disease Control and Prevention estimates that 6 in 10 Americans live with at least one chronic disease [1]. Chronic diseases like diabetes, heart disease, cancer, and stroke are the leading causes of death, disability, and increased healthcare costs in the United States and are largely managed through medication therapy. The prevalence of chronic disease and the shortage of primary care physicians continue to grow. This provides opportunities for community pharmacists to fill clinical gaps in care for patients [2]. Community pharmacies are among the most accessible healthcare locations in the nation. According to the National Association of Chain Drug Stores, roughly 9 out of 10 Americans live within five miles of a community pharmacy [3]. That distance shrinks to less than two miles for those living in metropolitan areas [4]. Ready access to community pharmacies in urban areas and their routine placement in rural locations position community pharmacies to be at the forefront of improving patient-centered outcomes for individuals with chronic diseases.

The traditional business model of the community pharmacy is historically based on medication product reimbursement. However, declining reimbursement for medication dispensing is creating a pressing need to accelerate the provision of patient-centered care [5]. Pharmacies across the United States have formed clinically integrated networks (CINs) to facilitate the provision of patient-centered care in community pharmacy locations. Member pharmacies of these CINs coordinate patient care and optimize medication use in collaboration with patients’ health care teams [6]. One of these networks, the Pennsylvania Pharmacists Care Network (PPCN) is a statewide CIN of over 150 community pharmacy locations across the Commonwealth of Pennsylvania [6,7]. As of June 2019, the PPCN is leading the nation as a statewide CIN with one of the highest numbers of pharmacy locations and active payor contracts for the provision of patient care services [6,8]. 

One goal of the PPCN is to leverage the pharmacy teams and geographic range across Pennsylvania to secure additional payor contracts. In addition to achieving critical geographic penetration, payors also want evidence that the patient care services provided by community pharmacists positively impact their members’ health outcomes [9]. Community pharmacy CINs like the PPCN need to continually innovate and demonstrate the value they bring to patients and the health care system. Performing systematic evaluations of practice innovations, interventions, and outcomes that are both robust in design and sufficiently powered can help CINs demonstrate their value. One way to accomplish these evaluations is to develop and formalize a practice-based research network (PBRN) [10]. PBRN formation can be leveraged from existing CINs like the PPCN. CINs can work in partnership with academic institutions to facilitate the research network formation with the goal of conducting outcomes research.

In August 2018, faculty and staff from the University of Pittsburgh School of Pharmacy received a Eugene Washington Engagement Award from the Patient-Centered Outcomes Research Institute (PCORI) to gather stakeholder input to develop a PBRN, the Pharmacy Innovation Network, comprised of Pennsylvania community pharmacies. One goal of this PBRN is to conduct rigorous outcome evaluations of pharmacist-provided care, including the patient care delivered through PPCN’s clinically integrated network [11]. PCORI’s mission is to support patient-centered outcomes research (PCOR) that helps patients make decisions about their desired health outcomes [12]. The Pharmacy Innovation Network is committed to doing PCOR in partnership with its pharmacy and patient stakeholders. National funding agencies, including PCORI, the Agency for Healthcare Research and Quality, the Robert Wood Johnson Foundation, and others are currently funding clinical research that focuses on patient-centered outcomes [13,14,15,16]. Funding organizations continue to drive the research landscape by supporting PCOR studies. Accordingly, there will need to be new ways to reach patients in the communities where they live to engage them in PCOR [17]. Pharmacists at community pharmacy locations across Pennsylvania may be able to reach and engage patients in PCOR in the communities where they live. The objective of this study was to learn the opinions of Pennsylvania pharmacists on participating in PCOR, including their perceived benefits, topics of interest, and engagement preferences. 

## 2. Materials and Methods

This survey research was conducted between January and April 2019. Pharmacists who were members of the Pennsylvania Pharmacists Association were eligible for inclusion. A 10-member Stakeholder Advisory Board (SAB), consisting of researchers, pharmacists who practice in PPCN member pharmacies (PPCN pharmacists), and patients was formed to provide input to the general survey content, wording, and flow. This partnered-approach to designing the survey was used to add external validity to the study. The SAB recommended that the survey gather information on (1) pharmacists’ readiness and capacity to participate in research and (2) their communication and engagement preferences. Draft survey questions were developed by the research team based on both feedback from the SAB members and a comprehensive review of existing literature on community pharmacy PBRNs. This draft survey was presented back to the SAB for additional feedback. SAB members provided recommendations on question and response addition, deletion, phrasing, and order. They also provided suggestions for how the survey could best be deployed. The final 20-item survey consisted of one multiple-answer multiple choice question and eight three-point Likert scale, eight open-ended, and three single response questions. Based on stakeholder suggestion, a brief 1-min video which defined PCOR and briefly explained how PCOR might occur in community pharmacies was created. This video was added to the beginning of the survey to help orient pharmacists to the overarching survey topic. Our advisory board felt this was important to include as most pharmacists would not have experienced PCOR in their pharmacies and would be at a disadvantage when answering survey questions.

The survey was deployed both electronically and on paper. Paper surveys were distributed at the Pennsylvania Pharmacists Association (PPA) Mid-Year Conference in January 2019 during an open general session for its members. Survey deployment at this conference was suggested by the SAB because it is the largest gathering of community pharmacists., in Pennsylvania. Many pharmacists who attend the meeting are also members of PPCN and may be interested in participating in PCOR in the future. The pre-survey video was shown to conference attendees and all pharmacists in attendance were then invited to take the survey. Responses from the paper surveys were entered into a Qualtrics^TM^ Survey Platform by a member of the research team. A survey link was also sent electronically to members of the PPCN twice in February and March 2019. An additional survey link was sent three times to PPA members in April 2019 through PPA’s regularly scheduled email newsletters. The survey was also highlighted during a PPCN teleconference. Pharmacists who completed the survey were offered the option to enter a drawing for one of three $100 prepaid debit cards. 

Descriptive statistics were used to analyze quantitative survey responses using IBM^®^ SPSS^®^ Statistics (Version 25; IBM Corp., Armonk, NY, USA). Chi-square tests were conducted to evaluate responses of PPCN pharmacists compared to other pharmacists. Percentages are reported based on total of number of respondents for each question. A content analysis of open-ended questions was conducted by the research team using qualitative methods [18]. This research was classified as exempt by the University’s Institutional Review Board.

## 3. Results

A total of 73 pharmacists responded to the survey and their demographics are provided in Table 1. Most respondents (47%) practiced in independent pharmacies, with supermarket, mass merchandiser, and traditional chain pharmacies representing a combined 24% of respondents. 

Nearly half (48%) of the respondents’ pharmacies were members of the PPCN and 44% received post-graduate residency or fellowship training. When asked about their interest in participating in research, 96% (n = 70) of pharmacists reported they wanted to hear more about research opportunities. Additionally, 95% (n = 69) believed that at least some of their patients are interested in PCOR. 

### 3.1. Perceived Benefits of Participating in PCOR

Pharmacists believe that “improving their patients’ health care goals” is the most important benefit to their patients for participating in PCOR. Eighty-six percent (n = 63) of respondents ranked this as very important to their patients. Additionally, 78% (n = 57) of respondents felt that “providing their patients with access to more health care resources” was also very important. Providing compensation to patients for research participation was not perceived to be as important as health care-related benefits.

**Figure 1:**
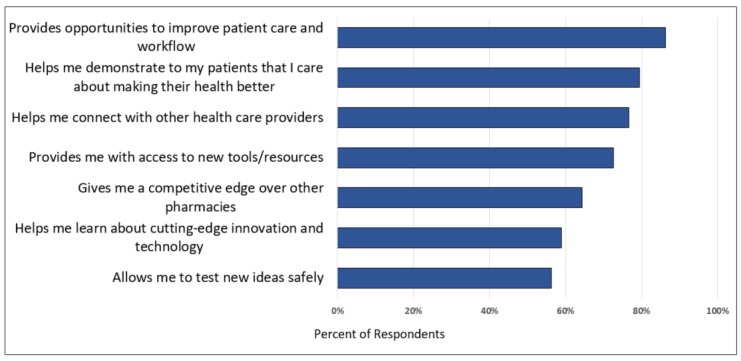
Pharmacists perception of the most valuable benefits from participation in patient-centered outcomes research for their pharmacy (n = 73).

Figure 1 describes pharmacists’ perceived benefits to their pharmacy practice for participating in PCOR. Improving workflow and patient care was reported as the most valuable benefit (86%, n = 63). Notably, nearly all PPCN pharmacists (34 of 35 respondents) described this benefit as very valuable compared to non-PPCN pharmacists (*p* = 0.035). Compared to non-PPCN pharmacists, a majority of PPCN pharmacists (80% versus 50%; *p* = 0.024) also believed participating in research would be very valuable in giving them a competitive edge over other pharmacies. The next most valuable benefits reported by all respondents included helping pharmacists demonstrate to patients that pharmacists care about making patient health better (80%, n = 58) and connecting pharmacists with other health care providers (75%, n = 55). 

### 3.2. Research Topics of Interest

**Figure 2:**
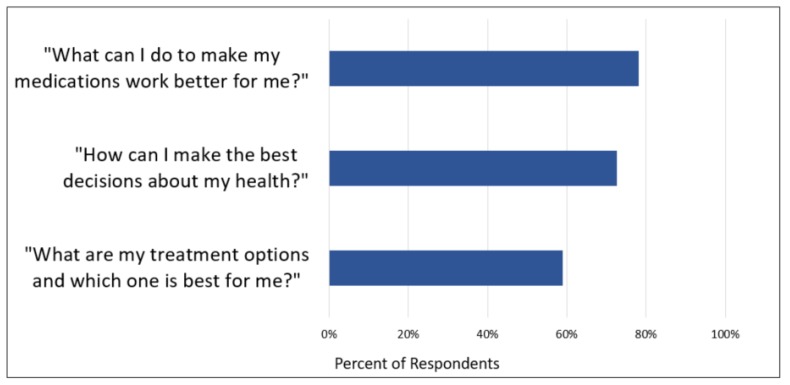
Patient-centered outcomes research questions rated as “very important” by pharmacists (n = 73).

Pharmacists were asked to rate a variety of research topics, including PCOR topics, that were of most interest to them. (see Table 2). Most pharmacists (81%, 58 of 72) expressed interest in researching pharmacist-prescriber collaboration. Medication costs was also a topic of high interest (76%, 54 of 71). Additionally, pharmacists were asked to rank their interest in answering three broad PCOR research questions their patients may ask. The percentage of pharmacists rating each question as “very important” is reported in Figure 2. 

Finally, a content analysis of the open-ended research topic questions revealed additional PCOR topics pharmacists were interested in which included: (1) increasing patient access to care; (2) enhancing patient transitions of care between healthcare settings; and (3) discovering patients’ internal motivators to increase engagement in their own care.

### 3.3. Patient Engagement Preferences

Pharmacists reported their preferred ways to engage patients in PCOR would be through face-to-face interactions at the pharmacy (76%, n = 55) and utilizing student pharmacists or interns to assist in the patient engagement process (72%, n = 52). Additionally, pharmacists provided the following ideas as options for engaging patients in PCOR: (1) utilizing pharmacy dispensing systems to alert pharmacists to patients who are eligible for PCOR studies; (2) organizing community outreach events; (3) collaborating with local providers to create referral processes; (4) creating a website for patients to receive more information about studies; and (5) using technology (kiosks, text messaging, and tablets) at the pharmacy.

### 3.4. Resources

Having a support person or other resources to assist with research tasks (85%, n = 62) and time during the workday to help with research (84%, n = 61) were also very important to the survey respondents. Pharmacist and patient compensation were viewed as less important to respondents. Respondents reported their top four preferences for learning and communicating about research opportunities were through email (85%, n = 62), professional organizations (59%, n = 43), a designated research network website (49%, n = 36), and webinars (42%, n = 31).

## 4. Discussion

The objective of this study was to learn Pennsylvania and CIN pharmacists’ opinions, regarding participation in PCOR, including perceived benefits, topics of interest, and preferred engagement methods. These findings will be used to facilitate development and growth of the Pharmacy Innovation Network, the first community pharmacy PBRN leveraged from an existing community pharmacy CIN. The goal of this PBRN will be to answer questions that are important to patients while demonstrating the value pharmacists bring to patients, payers, and the health care system. In the Pharmacy Quality Alliance’s action guide entitled, “Strategies to Expand Value-Based Pharmacist-Provided Care,” improving patient outcomes is a key component for the transition from fee-for-service to pay-for-performance in pharmacy practice [5]. Community pharmacy PBRNs, like our Pharmacy Innovation Network, provide an excellent opportunity to conduct outcome evaluations of pharmacist-provided care. These evaluations can facilitate transformation to value-based care. Indeed, pharmacists readily recognize that evidence is needed to support practice advancement in the community setting [19,20]. Since clinically integrated networks of community pharmacies are forming in nearly every state across the United States, there is ample opportunity for more community pharmacy PBRNs to grow from these networks and assist with outcomes research [6].

Engaging stakeholders to participate in a community pharmacy PBRN is essential to its success. In order to do this effectively, it is important to understand pharmacists’ motivations for research participation. Our survey respondents reported providing opportunities to improve patient care and pharmacy workflow as well as demonstrating a commitment to improving their patients’ health were the top benefits for participating in research. Previous research shows pharmacist commitment to patient health is an important motivator for community pharmacist participation in research [21,22]. With changes in drug reimbursement, community pharmacies need to find new ways to distinguish themselves. Participating in a PBRN is one possible avenue to demonstrate value-added care to patients. Research conducted through a PBRN can help pharmacies identify and scale best practices and support rapid innovation and implementation of services. Notably, PPCN pharmacists were significantly more likely than non-PPCN pharmacists to believe that participating in research would give them a competitive edge. This finding may be because most PPCN pharmacists practice at independent pharmacies that are seeking out innovative business models to provide patient care services in their communities. 

This study also showed pharmacists value resource support and time allocation for research activities more than they value compensation for participating in research. This aligns with previous research, which reported time constraints to be a significant barrier for pharmacist participation in research [19,22,23,24]. One potential solution to alleviate time constraints is to leverage student pharmacists in the research process. Nearly three-fourths of pharmacists felt students could assist with engaging patients in the research process. Pharmacists overwhelmingly felt face-to-face interaction, which already occurs as part of the pharmacists’ patient care process, is the best way to approach and engage patients in PCOR. A recent public opinion poll by CVS Health found that 69% of respondents visited their pharmacy at least once per month [25]. These touch points provide numerous opportunities for pharmacists and student pharmacists to engage their patients face-to-face in research. 

To develop the Pharmacy Innovation Network, we built upon previous experiences of other community pharmacy PBRNs [21,26]. One unique aspect in developing our network was the application of PCORI’s philosophy to engage patients and stakeholders as equitable partners in the research process [27]. We formed the SAB comprised of key pharmacist and patient stakeholders to guide the survey’s development, deployment, and analysis. This process also served as an implementation strategy to foster stakeholder interrelationships for the formation of the PBRN [28]. For example, the formation of the SAB helped the research team identify pharmacist champions to promote the Network and recruit colleagues to join the Network moving forward. We feel this implementation strategy was a vital step to form key relationships which will assist with further Network growth, strategic planning, and operations. As part of this PCORI Engagement Award, the research team will conduct pharmacist stakeholder interviews and patient focus groups to augment these survey findings and further refine the PBRN’s strategic plan.

This survey research comes with a few limitations. First, due to the survey distribution methodology agreed upon by our advisory board, we were unable to determine a survey response rate. Given that this survey was distributed through professional pharmacy organizations and networks, a selection bias may be present. The survey was distributed this way at the suggestion of our advisory board because pharmacists who are highly engaged in professional organizations are also the most likely to participate in a PBRN. Additionally, with 73 total responses, our findings may not accurately represent the opinions of pharmacists across Pennsylvania. Since we did not solicit information on pharmacist location, we were unable to determine whether the respondents represented all geographic regions of Pennsylvania. Another limitation is that the majority of our respondents were from independent community pharmacies. Although independent community pharmacies make up the vast majority of PPCN pharmacies, the Network plans to include pharmacies of all types. 

## 5. Conclusions

A stakeholder-driven survey of pharmacists was utilized to inform the development of a community pharmacy PBRN in Pennsylvania, the Pharmacy Innovation Network. Pharmacists are interested in research opportunities that could improve their workflow, patient care, and ultimately their patients’ health care goals. Face-to-face interactions were identified as the preferred way to engage patients in PCOR, and pharmacists felt student pharmacists can assist in this process. This research also confirmed previous findings which demonstrate pharmacists value both resource support and time allocation for research activities. The methods for survey development, deployment, and analysis included engagement of a stakeholder advisory board consisting of pharmacists and patients. This advisory board process also served as an implementation strategy to facilitate stakeholder interrelationships for the formation of the PBRN. These results can inform others who are actively structuring processes and developing communication strategies for forming community pharmacy PBRNs, especially in partnership with CINs.

## Figures and Tables

**Table 1:** Survey respondent demographics (n = 73 *).

Characteristic	Value
Average age in years (range)	42 (24–79)
Average number of years post-graduation (range)	18 (1–53)
Participants with residency or fellowship training, n (%)	32 (44%)
Practice Setting, **n (%)**	
Independent pharmacy	34 (47%)
Traditional chain pharmacy	8 (11%)
Supermarket chain pharmacy	9 (12%)
Mass merchandiser pharmacy	1 (1%)
Other	20 (27%)
Pennsylvania Pharmacists Care Network Member Pharmacy	35 (48%)

* Demographic information was reported voluntarily by survey participants. Not all requested information was provided by all participants.

**Table 2:** Research topics of most interest to pharmacists.

Research Topic	Number (%) ^
Prescriber Collaboration	58 (81%)
Medication Costs	54 (76%)
Innovative Patient Care	47 (65%)
Immunizations	47 (65%)
Expanding Business Models	42 (58%)
Adherence Packaging	39 (54%)
Technology	35 (49%)
Medication Synchronization	33 (46%)

^ Percentages represent proportion of respondents for each item.
